# LITHORISK.COM: the novel version of a software for calculating and visualizing the risk of renal stone

**DOI:** 10.1007/s00240-020-01228-0

**Published:** 2020-11-27

**Authors:** Martino Marangella, Michele Petrarulo, Corrado Vitale, Piergiuseppe Daniele, Silvio Sammartano

**Affiliations:** 1 Fondazione Scientifica Mauriziano Hospital, Turin, Italy; 2 Kidney Stone Laboratory, Mauriziano Hospital, Turin, Italy; 3 Nephrology and Dialysis Unit, Mauriziano Hospital, Turin, Italy; 4 Department of Analytical Chemistry, University of Turin, Turin, Italy; 5 Department of Inorganic Analytical and Physical Chemistry, Messina, Italy

**Keywords:** State of saturation, Soluble complex species, Stability constant, Calcium oxalate, Brushite, Struvite, Cystine, Uric acid

## Abstract

Estimation of state of saturation with stone-forming salt represents a reliable tool to assess the overall risk. The available methods are based on computer-assisted ab initio calculations. Our earlier method URSUS was subsequently substituted by Lithorisk®, a software including visualization of risk profiles. Unfortunately, Lithorisk does not adapt to new versions of Windows® and Macintosh® Apple, neither runs on smartphones or tablets. We propose a novel version of the software which can be directly used online on any device equipped by different operating systems. Upon online connection and after registration, the software is ready for unlimited accesses, in either Italian, English or French. After digiting input variables (urea and creatinine also included) in a fixed dashboard, state of saturation is promptly given. In addition to state of saturation (ß) with calcium oxalate, brushite and uric acid, ß struvite and cystine are available. Both input variables and ß results are graphically depicted as green or red horizontal bars to indicate recommended values. The software was implemented with equations allowing to omit sulphate and ammonium excretion for users with difficult access to these measurements. This simplified version, tested for ßCaOx and ßBsh on 100 urine samples showed close correlation with the full version. The software gives a list of total and free concentrations and soluble complex species distribution. Results can be printed or saved as PDF. So, we propose an easily accessible software to estimate state of saturation usable on any operating system and personal device.

## Introduction

The essential condition for a renal stone to form is supersaturation of urine with respect to lithogenic salts [1–3]. While supersaturation fully defines some types of stone disease, i.e., cystine, uric acid, struvite stones, it is not sufficient to explain calcium containing renal stones. In this subset, stones are thought to result from imbalance between promoters and inhibitors of crystal formation [4]. Therefore, in addition to thermodynamic processes, well described by the state of saturation, kinetic processes including nucleation, growth and aggregation, role of macromolecules and their effect on crystal cell interaction in renal tubules, are relevant for crystallization [5, 6]. However, despite extensive experimental studies on kinetics of crystallization, the methods proposed so far are complex, time consuming and not standard controlled. This is why a recent Consensus Conference did not recommend their use in clinical practice [7]. This also applies to so-called semi-empirical methods for estimating urine saturation which, therefore, are not extensively used [2]. Conversely, the measurement of the relative supersaturation, based on computer calculations, was shown to represent a simple and useful tool to manage patients and improve compliance to preventive treatment as well [8–14].

Years ago, we had developed our own method for calculating urine saturation with stone-forming salts, called URSUS and distributed as CD-ROM in our country [15]. This method used computer-assisted ab initio calculations to estimate the relative saturations (ß) for calcium oxalate (CaOx), brushite (Bsh) and uric acid (UA). While input variables were the same as those used by others, instead of *thermodynamic* stability constants, *conditional* stability constants of soluble complex species, reassessed at actual ionic strengths, were used [11]. To better support users in the interpretation of results, we subsequently produced Lithorisk ®, a software for both calculation and graphic visualization of risk profiles for stone formation [16]. However, this software cannot be changed so as to adapt to upcoming new versions of Windows®, nor it can work on Macintosh® Apple computer or run on smartphone or tablets. Therefore, we have developed a new software which can be directly used online on any device equipped by different operating systems. The software has been improved in that it now provides a reliable estimate of ßCaOx and ßBsh even if sulfate and ammonium measurements are not available. Also, the state of saturation with both struvite and cystine has been added in the calculations.

## Materials and methods

The source code has been completely re-written based on the original files [11]. Despite some difficulties, we were able to simplify the use of the program by disposing an online connection. The interface presentation and the graphical output have been changed. The software cannot be downloaded but can be accessed directly online upon registration as a new user, and used on a personal device in either Italian, English or French language.

Each input variable must be digited in a fixed dashboard in the preferred units. If urine volume is available, input values, given as 24-h urine excretion, are automatically transformed as concentrations per liter. At any value, a green or red horizontal bar will correspond, to evidence normal or abnormal values according to their positive or negative effects on supersaturation, respectively. The sense of the effect conforms to arbitrary though widely accepted reference ranges, which are delimited by a thin yellow bar [2, 17–20]. Upper and lower limits for input data have been set to avoid erroneous input and will not be accepted by the software. At the end of calculation, patient’s personal information can be added. To fulfill the privacy rules and assure safeness of the data, the measurements and results cannot be saved online, but can be printed or saved as PDF file on personal devices.

## Results

**Fig. 1:**
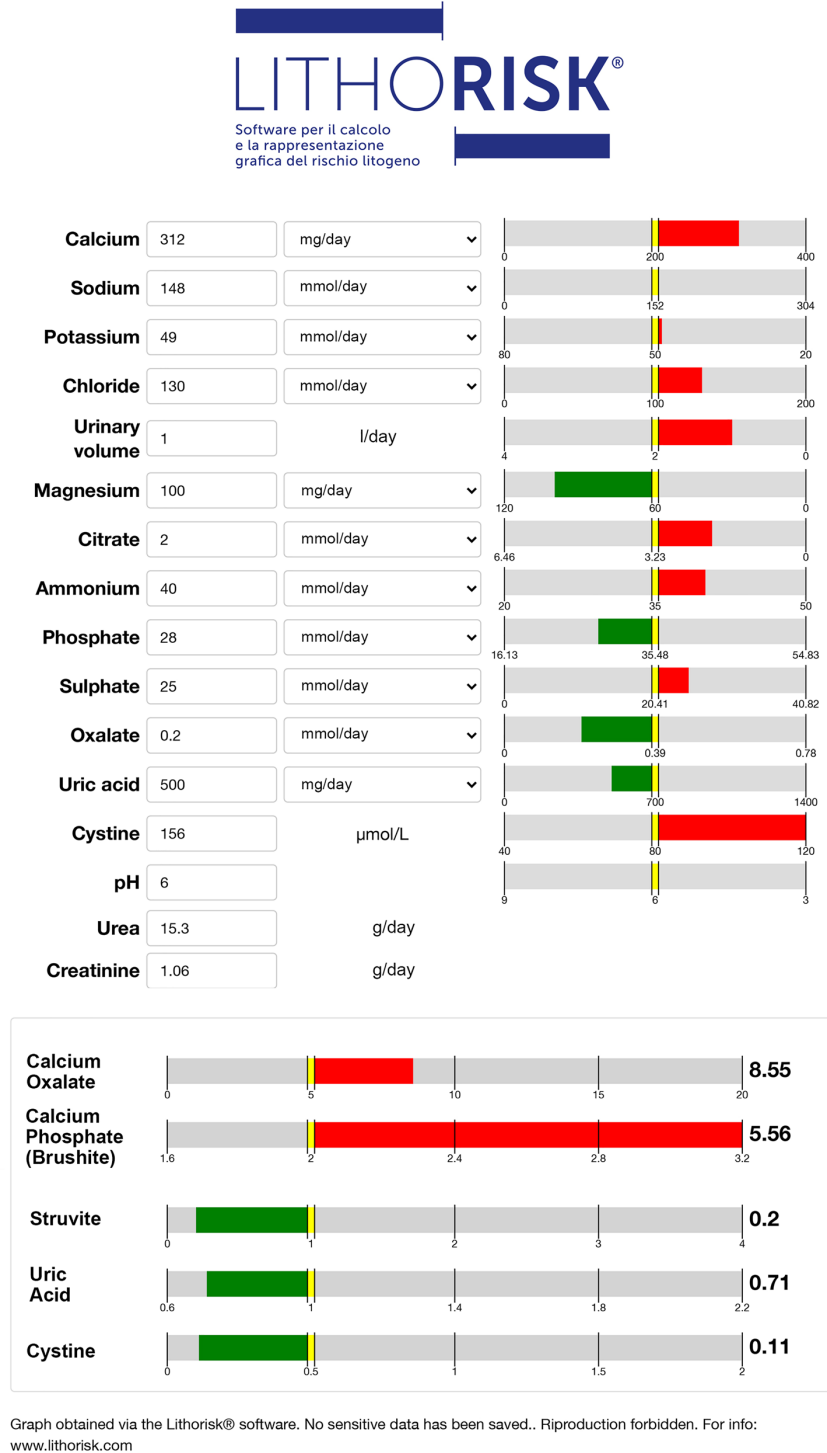
Illustration of the dashboard filled with measurements from a sample 24 h urine. Units can be changed from mg to mmol and viceversa according to usual laboratory reports. Green or red bars indicate whether or not measurements are within a recommended arbitrary range. Results of the calculations of state of saturation with calcium oxalate, brushite, struvite, uric acid and cystine are shown in the bottom of the figure. Green or red bars indicate whether ß values are within a recommended arbitrary range. Patient’s personal data can be given

Figure 1 illustrates and exemplifies a dashboard filled with input variables necessary to make one sample calculation. Both excretions of urea and creatinine are required. Urea measurements provide information about dietary protein intakes, whereas creatinine can be used to both normalize different parameters and verify correctness of urine collection. Other diet-related parameters are sodium, potassium and sulfate excretions. Inputs can be given in different units (mmol/L or mg/dL) conforming to the current laboratory reports. Upon completion of data entry, results of state of saturations will be given upon clicking on the bottom bar.

**Fig. 2:**
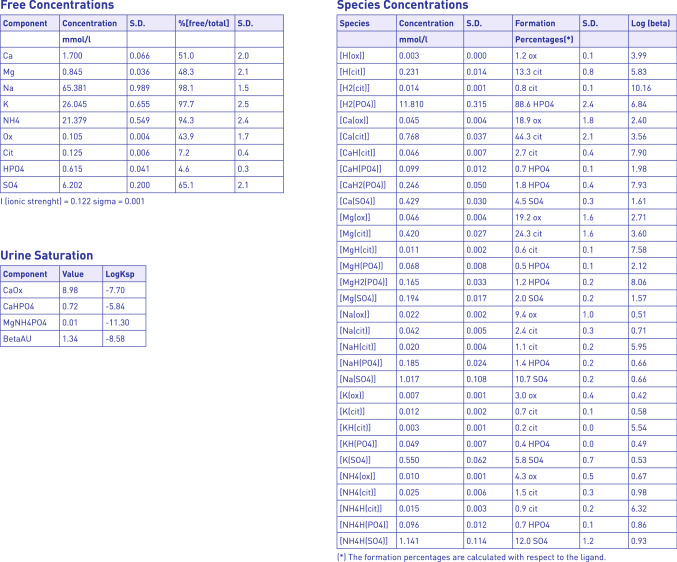
Species distribution in urine from a single patient, are displayed after clicking of the Details bar (not shown in figure). The whole record can be printed or saved on personal device

Results of ß which promptly appear are depicted as horizontal bars, green in case of ß < 1 for uric acid and struvite. Thresholds of normal range for calcium oxalate, brushite and cystine, denoted by a small yellow bar, are of course arbitrary, and refer to recommended levels of saturation emerging from the literature [8, 9, 16–22]. The sheet can be completed with personal information of the patients and finally can be printed or saved as PDF. In addition, by clicking on the Details gray bar, it is possible to get a list of total and free concentrations and soluble complex species distribution as well (Fig. 2). This option can be of particular interest for expert clinicians and basic scientists to be used for research purposes.

Many potential users had complained to be unable to use the first version of Lithorisk® because their laboratories did not supply measurements of inorganic sulphate and urinary ammonium. To obviate this drawback, in this last version, the software was implemented with equations, whereby estimates of the above components were derived, by means of multiple regression analysis, from measurements commonly available everywhere. Regression analysis was performed by using a previous database [23]. From this, we found that urea and phosphate were reliable predictor for sulphate and urea and creatinine for ammonium, according to the following equations (all parameters as mmol/24 h):

1 $$\begin{gathered} {\text{Inorganic Sulphate}}\, = \,0.0{\text{43 Urea}}\, + \,0.{\text{125 Phosphate }}{-}{1}.{53,} \hfill \\ r^{{2}} \, = \,0.{76}\;p\, < \,0.000{1}\;n\, = \,{233;} \hfill \\ \end{gathered}$$

2 $$\begin{gathered} {\text{Ammonium}}\, = \,0.{\text{215 Creat}}\, + \,0.0{\text{54 Urea}}\, + \,{13}.{1,} \hfill \\ r^{{2}} \, = \,0.{29}\;p\, < \,0.000{1}\;n\, = \,{233}{\text{.}} \hfill \\ \end{gathered}$$

**Fig. 3:**
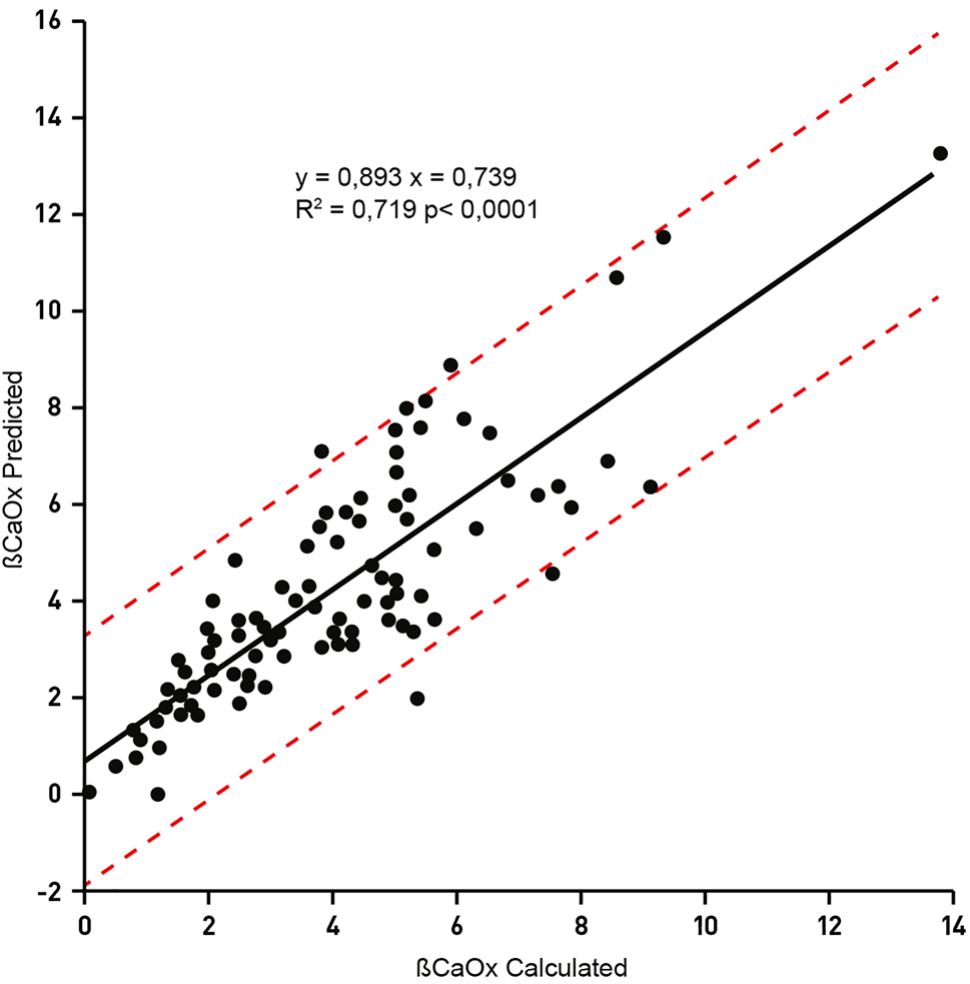
Correlation between measurements of calcium oxalate relative saturation (ßCaOx) carried out using sulphate and ammonium excretions either measured or derived by regression analysis reported in Eqs. (1) and (2). Correlation includes 100 urine samples. Dotted lines indicate 95% confidence intervals

**Fig. 4:**
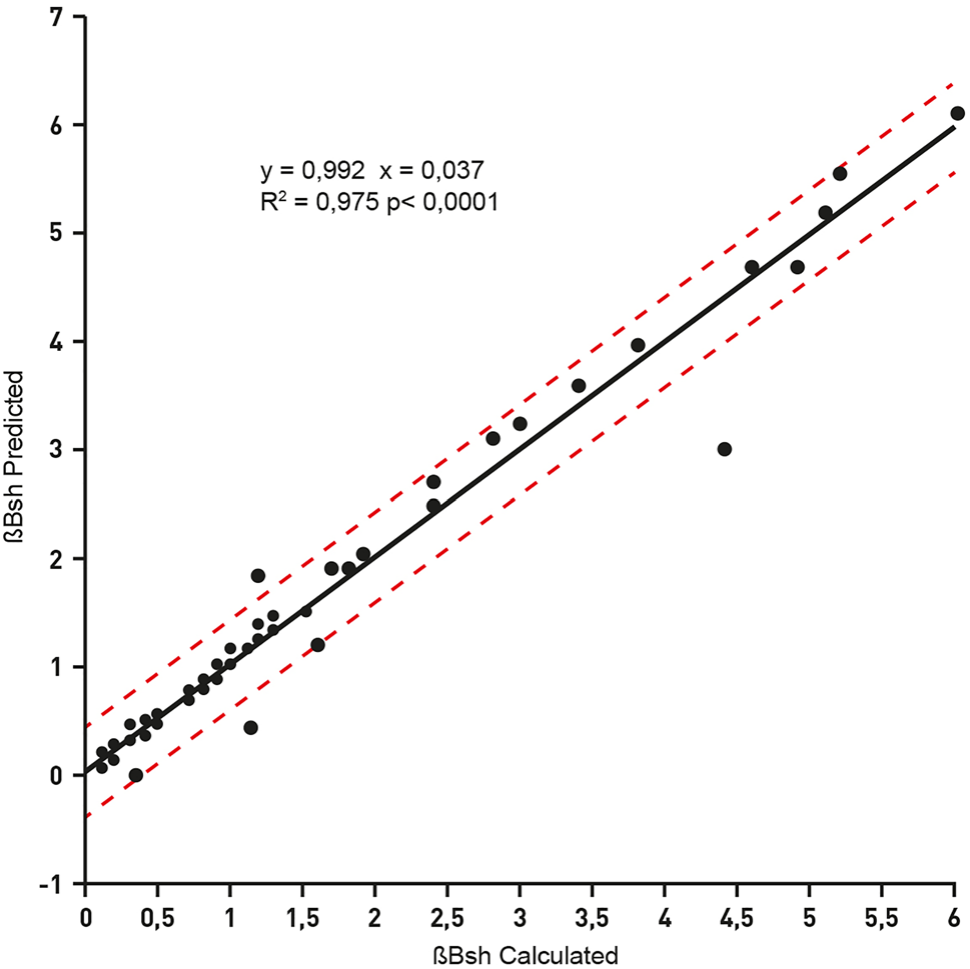
Correlation between measurements of brushite relative saturation (ßBsh) carried out using sulphate and ammonium excretions either measured or derived by regression analysis reported in Eqs. (1) and (2). Correlation includes 100 urine samples. Dotted lines indicate 95% confidence intervals

To test the reliability of the above equations, we calculated ßCaOx and ßBsh using both actually measured and predicted values of sulphate and ammonium. The correspondence between the two sets of measures made on 100 urine collections appeared quite satisfactory as evidenced in Figs. 3 and 4.

## Discussion

Nephrolithiasis is a disease with a great epidemiological impact, since up to 10% of the general population is expected to form at least one stone in their lifespan [24] and, in a considerable part of cases, it recurs [25]. This disease can be successfully managed by integrating urological and medical approach, the latter aimed at reducing stone recurrence rate [26]. Metabolic evaluation stands as a crucial means to assist physicians in patients’ management [7, 19, 20]. Despite extensive studies, much remains to understand about pathogenesis of stone disease, at least concerning calcium nephrolithiasis. An imbalance between saturation and inhibition in urine environment was suggested as a crucial risk factor [4]. While studies on inhibitors are still weakened by a number of theoretical and practical flaws, measurements of state of saturation have widely spread, appearing as an easy and reliable tool. It is well accepted that state of saturation cannot be used to distinguish between stone formers and non-stone formers, nor between single and recurrent stone formers, in that a wide overlapping of data has been found in virtually all saturation studies [11, 21, 27]. As a matter of fact, while measurements of excretion of urine components inform us about derangements in metabolic pathways and dietary disorders, state of saturation gives a view of the propensity for crystals to form with the consequent risk of stones.

Some contend that to achieve saturation estimates requires that many urinary components be measured with the consequent increase in cost. This explains why several urine-based indices have been proposed trying to simplify the assessment of the stone risk. These include the Tiselius Index [28], the Robertson PSF index [29], the Bonn-Risk Index [30], the upper limit of osmolality proposed by Porowski et al. [31] and maybe many others. However, none of these has widely diffused, so that they warrant confirmation by clinical trials.

Herein, we report a new version of the software Lithorisk® which is characterized by several novelties:

1. There is a direct access to the software through web on users’ devices, including PC and laptop, tablets and smartphones, independently of the operating system. The software is based on the earlier version [16] and uses the same stability constants of soluble complexes handled with the same math principles.
2. There is a completely renewed graphic presentation.
3. It is now possible to get estimates of struvite and cystine saturations.
4. Species distribution is available, including concentration and percentage of both cations and anions and soluble complexes as well. In expert hands, this could be an important option to evaluate the impact of varying ion concentrations on species distribution to optimize effects on state of saturation. For instance, it may be relevant to assess effects of different citrate and magnesium concentrations on ionic equilibria and proton concentration [22].
5. The new software is equipped with subroutines that generate ß values even omitting the sulphate and ammonium concentrations. Despite their marginal influence on the calculations, sulfate and ammonium determinations are generally included in ab initio calculations, including ours. Indeed, changing concentrations from the lower to upper limits of normal distribution, the effects become not irrelevant. For instance, changing sulphate concentration from 10 to 40 mmol/L, leaving other components unchanged, results in 22% variation of ßCaOx. The correlation of Eq. (2) (see “Results” session), concerning ammonium, is not much close. However, this impacts only marginally the calculations of ßCaOx and ßBsh because of the weak stability constant of ammonium with corresponding ligands in urine [11]. Of course, ammonium may have greater influence on ßStruvite, and in this case, we do not recommend to utilize the simplified version that uses calculated and not measured ammonium concentrations.

To ease the task of producing this version of Lithorisk®, it was decided to maintain the original list of soluble complexes. This may suffice in the routine management of patients, namely, to improve and control their compliance to treatment [7]. In addition, the prompt visualization of the results allows to simulate beneficial or detrimental effects on the stone risk generated by changes in urine chemistry and volume. For instance, the software can be used to tailor values of urine volume or calcium or other components that will result in safer saturation levels in individual patients. Therefore, this new program is essentially similar to previous others that measure state of saturation, including our previous one. We had reported a nice correspondence between our results and those obtained with EQUIL 2 [11]. The program has not been compared to the more recent JESS [13. 14] which includes mixed calcium-citrate–phosphate species and has been reported to yield different results from previous ones at least at higher urine pHs [32]. Work is in progress to assess whether integrating our program with the above complex species, or weak urea complexes [33], or phytate [34], will affect results in a significant way.

In conclusion, the essential novelty of this software is that it can be easily available for use in any operating systems and personal devises. Also, the great simplification of the input variable procedure, including the option to change units, and the novel graphical visualization of risk profiles are likely to ease the use and widen the understanding of the concept of saturation in the general practice of stone disease.

## Data Availability

Biohealth Italia srl Lithocenter data base.
